# Development of an evidence-based brief ‘talking’ intervention for non-responders to bowel screening for use in primary care: stakeholder interviews

**DOI:** 10.1186/s12875-018-0794-6

**Published:** 2018-06-30

**Authors:** Debbie Cavers, Natalia Calanzani, Sheina Orbell, Gabriele Vojt, Robert J. C. Steele, Linda Brownlee, Steve Smith, Julietta Patnick, David Weller, Christine Campbell

**Affiliations:** 1 The Usher Institute of Population Health Sciences and Informatics, Medical School, University of Edinburgh, Doorway 1, Teviot Place, Edinburgh, EH8 9AG UK; 2 Department of Psychology, University of Essex, Wivenhoe Park, Colchester, CO4 3SQ UK; 3 Department of Psychology, Social Work and Health Sciences, Glasgow Caledonian University, 70 Cowcaddens Road, Glasgow, UK; 4 Division of Cancer Research, Ninewells Hospital and Medical School, Mailbox 4, Level 6, Dundee, DD1 9SY UK; 5 Scottish Bowel Screening Centre, Kings Cross Hospital, Clepington Road, Dundee, DD3 8EA UK; 6 NHS Bowel Cancer Screening Midlands and North West Programme Hub, St. Cross Hospital, Barby Road, Rugby, CV22 5PX UK; 7 Cancer Epidemiology Unit, Oxford University, Richard Doll Building, Roosevelt Drive, Oxford, OX3 7LF UK

**Keywords:** Cancer, Primary care, Behaviour change, Bowel screening, Intervention

## Abstract

**Background:**

Bowel cancer is the third most common cause of cancer death worldwide. Bowel screening has been shown to reduce mortality and primary care interventions have been successful in increasing uptake of screening. Using evidence-based theory to inform the development of such interventions has been shown to increase their effectiveness. This study aimed to develop and refine a brief evidence-based intervention for eligible individuals whom have not responded to their last bowel screening invitation (non-responders), for opportunistic use by primary care providers during routine consultations.

**Methods:**

The development of a brief intervention involving a conversation between primary care providers and non-responders was informed by a multi-faceted model comprising: research team workshop and meetings to draw on expertise; evidence from the literature regarding barriers to bowel screening and effective strategies to promote informed participation; relevant psychological theory, and intervention development and behaviour change guidance. Qualitative telephone interviews with 1) bowel screening stakeholders and 2) patient non-responders explored views regarding the acceptability of the intervention to help refine its content and process.

**Results:**

The intervention provides a theory and evidence-based tool designed to be incorporated within current primary care practice. Bowel screening stakeholders were supportive of the intervention and recognised the importance of the role of primary care. Interviews highlighted the importance of brevity and simplicity to incorporate the intervention into routine clinical care. Non-responders similarly found the intervention acceptable, valuing a holistic approach to their care. Moreover, they expected their primary care provider to encourage participation.

**Conclusions:**

A theory-based brief conversation for use in a primary care consultation was acceptable to bowel screening stakeholders and potential recipients, reflecting a health promoting primary care ethos. Findings indicate that it is appropriate to test the intervention in primary care in a feasibility study.

**Electronic supplementary material:**

The online version of this article (10.1186/s12875-018-0794-6) contains supplementary material, which is available to authorized users.

## Background

Bowel cancer is an important public health issue; it is the third most common cancer and the fourth most common cause of cancer death worldwide [1]. Screening has been shown to reduce bowel cancer mortality, but high levels of participation are required [2]. In Europe, national screening programmes are still being implemented across all EU member states following European Council recommendations for consistency in coverage and the type of test used [3]. In the UK, a rolled-out national programme in all constituent countries has been delivered centrally via regional hubs since 2010, where a guaiac-based Faecal Occult Blood test (FOBt) is sent by post to eligible patients (those aged 60–74 years; 50–74 in Scotland) every two years [4]. Overall participation is currently 57.5% (45.1% vs 66.5% for the most deprived and least deprived groups respectively) [5].

Policy initiatives in the UK have focused on the prevention and early detection of cancer (including screening and early diagnosis) in order to reduce overall mortality [6, 7]. Although bowel screening kits are sent straight from the regional hubs, and therefore primary care has no direct involvement in the delivery of bowel screening, there is increasing recognition that primary care is well placed to mediate between bowel screening programmes and the public [8]. As such, general practices are provided with a regular list of non-responders and, in Scotland, have been financially incentivised to increase bowel screening participation among their patients [9]. In this context, a number of bowel screening interventions have been trialed [10]. Interventions shown to be successful in increasing uptake include those using patient reminder letters [11] and General Practitioner (GP) endorsements of patient invitation letters [12]. Brief interventions have been effective in relation to alcohol consumption and smoking cessation in primary care [13, 14], but less is known about their use in relation to the topic of bowel screening.

We aimed to develop and refine a theory-driven brief ‘talking’ intervention for opportunistic use in primary care consultations with non-responders to bowel screening. The use of robust theories enhances intervention implementation and evaluation, helping to determine why an intervention was (or was not) effective [15] – indeed, theory-driven interventions have been tested widely in the field of screening, including strategies to increase bowel screening uptake [16, 17]. In this paper we describe the process of developing the brief intervention and a preliminary evaluation of its content and acceptability using qualitative interviews with bowel screening stakeholders and bowel screening non-responders.

## Methods

### Developing the Brief Intervention

**Fig. 1:**
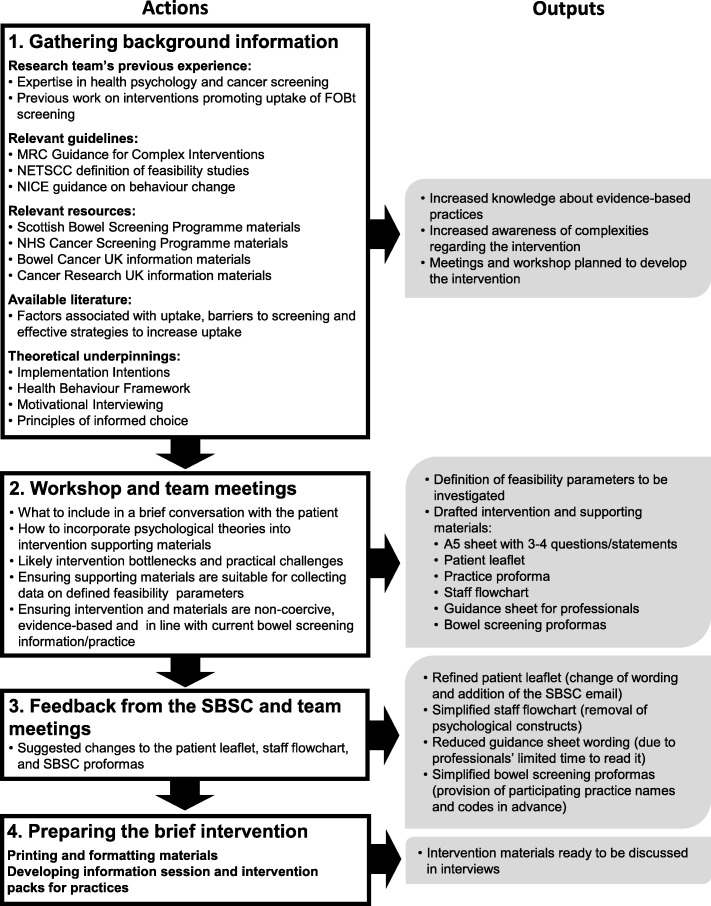
Developing the bowel screening brief intervention

The development process to design a theory-driven conversation between a primary care provider (PCP) and their eligible patient (a non-responder to their last bowel screening invitation) is illustrated by the flowchart (Fig. 1). Initially, relevant motivational and behavioural targets were identified based on our previous work on bowel screening interventions [18]. Existing evidence on factors associated with screening participation and effective strategies to improve participation was incorporated [10, 19]. The intervention drew on a variety of theoretical frameworks and relevant guidance. The second stage involved a workshop to develop supporting materials and identify implementation challenges. This included the identification of barriers or ‘bottlenecks’ when implementing each stage of the intervention such as the primary care provider having too many issues to deal with, the patient not being receptive to the intervention or the patient requesting a new kit but not ultimately completing it (Additional file 1: Intervention bottlenecks). Feedback on the draft materials was then requested from the Scottish Bowel Screening Centre (SBSC) and used to finalise them.

### Theoretical underpinnings

In developing our intervention we sought to draw on psychological theories commonly utilised in screening studies that target the active components of behaviour change. This approach allows an understanding of the barriers and incentives to changing behaviour; it can guide analysis, and it offers a consistent and transparent implementation framework that is generalisable across populations and health care settings [15]. The theoretical underpinnings include tenets from the implementation literature, motivational interviewing, the Health Behaviour Framework, and implementation intentions as well as guidance on complex interventions, behaviour change and informed choice that have been effective in previous work [20]. Further details on the included theory can be found in Additional file 2: Theoretical underpinnings.

### Consultation interviews

As part of the development and refinement process, one -to -one semi-structured telephone interviews were conducted with UK bowel screening stakeholders with a systems-level perspective of the screening landscape, and with potential intervention recipients (non-responders) to ensure a patient-centred approach. Interviews assessed the acceptability, and potential challenges of the intervention content and process before testing its feasibility in a primary care setting. Feedback therefore reflected the views of both those involved in delivering the intervention at the interface between public health and primary care, and those who would be eligible to receive it.

### Recruitment and Sampling

#### Stakeholder interviews

Bowel screening stakeholders were identified from around the UK and sampled to include senior staff within the national screening programmes in England, Northern Ireland and Wales, health board coordinators in Scotland, a lead cancer GP and a practice manager; representing a range of professional roles and geographical locations with varying screening uptake rates. Intervention supporting materials were shared with this group of interviewees.

#### Non-responder interviews

Non-responders were recruited via two bowel screening centres: the Scottish Bowel Screening Centre in Dundee (Scotland) and the Midlands and North West Bowel Screening Hub in Rugby (England). Potential participants were identified from records of non-responders to the most recent round of bowel screening invitations. Letters of invitation to interview, an information sheet detailing the study, and a response card with FREEPOST envelope were delivered to 500 non-responders by the NHS screening centre managers on behalf of the research team. Potential respondents were purposively selected based on age, gender, socio-economic status, geographical location (six Scottish health boards: NHS Lothian, NHS Tayside, NHS Lanarkshire, NHS Argyll and Clyde, NHS Shetland and NHS Orkney, and one large English region: The Midlands and North West), and number of times the kits were not returned, to represent a range of views and experiences. A total of 33 reply cards (6.6%) were returned to the research team, of which 24 were eligible for interview (the remaining nine turned out to be responders or were being followed up with regular colonoscopies). Individuals were offered a £15 high street shopping voucher.

### Analysis

All interviews were transcribed verbatim and anonymised. Transcripts were read and analysed using thematic analysis informed by a grounded theory approach [21, 22]. Open, line by line coding of the transcripts took place to stay close to the raw data and allow for any issues to be identified beyond those assumed significant by the researcher (DC). All were read and a proportion coded by three researchers. This was followed by focused and axial coding to formulate a structured set of codes rooted in and explained by the data. Constant comparison within and across transcripts took place as part of the coding process to iteratively develop and refine the coding framework. The transcripts were examined for contrasting or deviant cases and these were interpreted in the context of the data and through discussion within the research team.

## Results

### The Intervention

**Fig. 2:**
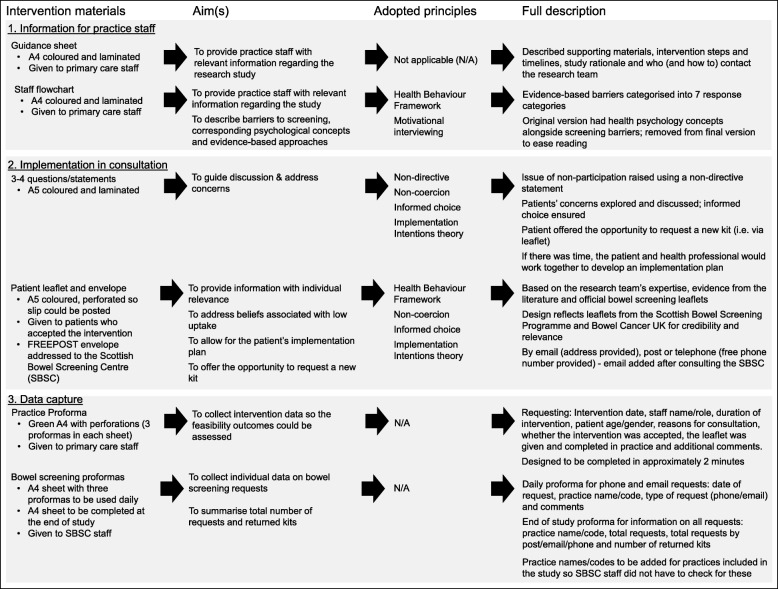
Intervention supporting materials

Our developmental process resulted in a ‘talking’ intervention, which comprised a brief guided conversation between a PCP and a patient non-responder attending a routine primary care consultation. The conversation was supported by a number of materials to inform and guide it (Fig. 2). These comprised 3–4 suggested questions the PCP could use to introduce the topic of non-response to a bowel screening invitation, a patient leaflet with an included implementation intention plan and details of how to request a new screening kit, a flowchart, and a guidance sheet for PCPs. Intervention proformas were also developed to be completed by 1) primary care staff after each intervention, and 2) SBSC staff after each kit request and summarising all kit requests.

In addition, a planned package of support was developed to help practices implement and sustain the intervention, including: a briefing session to provide background information on the intervention and its theoretical and evidential underpinnings; support to set up the intervention according to practice needs; and regular appropriate practice contact.

### Stakeholder interviews

Eight individuals were identified and invited to interview via email and telephone: two declined but suggested others who would be suitable. Seven semi-structured qualitative interviews were carried out by telephone and lasted approximately 30 min (see Table 1) (Additional file 3). Stakeholders are not named and details of their precise role are not included to protect confidentiality.

**Table 1:** Characteristics of bowel screening experts^a^

Pseudonym	Sex	Region
Bowel Screening Expert 1	Female	Lanarkshire, Scotland
Bowel Screening Expert 2	Female	Belfast, Northern Ireland
Bowel Screening Expert 3	Female	Rhondda Cynon Taf, Wales
Bowel Screening Expert 4	Female	Shetland, Scotland
Bowel Screening Expert 5	Male	Forth Valley, Scotland
Bowel Screening Expert 6	Female	Tayside, Scotland
Bowel Screening Expert 7	Female	Lothian, Scotland

^a^All participants occupied a senior position in their specific areas (Bowel Screening leads, Cancer leads, GP leads or practice managers)

Bowel screening stakeholders were involved in a number of local initiatives, sometimes linked to national campaigns, to increase uptake of bowel screening in their local area. Initiatives included working with general practices, public information campaigns, workshops to increase awareness among health professionals and work visits.

Themes identified in these interviews are: *the role of primary care, feedback on the intervention,* and *potential obstacles.*

### The Role of Primary Care

Bowel screening stakeholders recognised the significant role for primary care in promoting bowel screening uptake; PCPs provided a trusted source of information, support and encouragement for patients,

“I think the big involvement of general practice has probably contributed quite a lot to that increase in uptake [...] where you’ve got the GPs engaged and they are talking to patients opportunistically.” (Bowel screening stakeholder 1)

Stakeholders also mentioned the importance of multi-disciplinary working in the primary care context and the necessity to make sure PCPs had up to-date knowledge of bowel screening and bowel cancer to pass on to their patients. Targeting interventions for specific groups was also reported as important. Interviewees recognised that there would be variability between practices and it would be important to adapt to their existing practices and working style.

### Feedback on the Intervention

Bowel screening stakeholders were supportive of the intervention and its objectives: to promote a clear and coherent bowel screening message, raise awareness and normalise its practice, and discuss screening as part of a proactive approach to people’s own health,

“I thought actually it was a really good idea. I think it’s really important that there’s a consistent message from the professionals going out. […] I thought it was really straightforward and should be very effective.” (Bowel screening stakeholder 3)

A face-to-face approach was also considered more personal and thus more likely to meaningfully connect with individuals and feel relevant to them,

“Having that targeted and it might not be very much that’s needed on a personal level to, for somebody to decide they will go and do it.” (Bowel screening stakeholder 4).

The intervention was considered workable in a primary care setting and the wording was acceptable and non-coercive,

“I think they’re (the questions) good because they’re open questions they’re not direct…and they’re allowing the person to respond in a number of different ways; you’re not pushing down on one particular response.”(Bowel screening stakeholder 5)

Stakeholders also suggested particular opportunities for delivering the intervention, such as chronic disease or flu clinics, and stressed the importance of utilising the whole primary care team.

### Potential Obstacles

Potential challenges related to the time and resource-constrained primary care environment, the risk of overwhelming PCPs with too much paperwork and being too prescriptive, thus belittling their wealth of experience in patient communication and care.

The main reservation and anticipated obstacle was lack of time and competing priorities for health promotion,

“I think the GP will be stretched in their ten minutes they have or less […] to actually sort out their immediate problem […] They’ve also to look at other things like smoking, weight management, all these other things that they’re being bombarded with in terms of health messages; it’s how do they prioritise? Which of these things do they pick?” (Bowel screening stakeholder 2)

Reservations were voiced about the time it would take to address implementation intentions; although it was viewed as a helpful ‘stem’, participants indicated that the use of materials may vary across different PCPs. Additionally, stakeholders cautioned that it would be difficult to measure the impact of the intervention in the context of other ongoing efforts to increase screening participation.

### Non-responder Interviews

Twenty-four telephone interviews were conducted exploring experiences of being invited to take part in bowel screening, reported reasons for not completing the test, and responses to the proposed brief intervention scenario as described above, including the opportunity to request a new bowel screening kit (Additional file 4). Non-responders did not see the supporting materials for the intervention as these had not yet been piloted in primary care and thus shown to be acceptable. The intervention scenario was described to them in detail. Participants in five interviews were subsequently deemed ineligible due to ongoing bowel investigations, leaving 19 interviews in the final analysis (9 female, 10 male) (Table 2). Data on age were not obtained due to confidentiality issues. All interviewees were within the eligible age groups for screening (50–74 in Scotland and 60–74 in England), with two years added to the maximum age to include non-participants who were last invited at the age of 74 (invitations happen every two years).

**Table 2:** Characteristics of interviewed non-responders

Pseudonym	Sex	Region	Country	SIMD/IMD decile^a^	Regular GP attendance
Sarah	female	Greater Glasgow & Clyde	Scotland	10^b^	No
Bill	male	Lothian	Scotland	8	No
Andrew	male	Lothian	Scotland	8	No
Fred	male	Lothian	Scotland	not available	No
Maureen	female	Lothian	Scotland	6	No
Elsie	female	Lothian	Scotland	9	No
Joyce	female	Lanarkshire	Scotland	3	Yes, regular bloods
Sandra	female	Lanarkshire	Scotland	4	No
William	male	Lothian	Scotland	3	Yes
Angela	female	Lothian	Scotland	7	No
Flora	female	Lanarkshire	Scotland	2	No
Gregor	male	Lanarkshire	Scotland	9	Yes
Gavin	male	Greater Glasgow & Clyde	Scotland	10	Yes
Gillian	female	Tayside	Scotland	5	No
Alan	male	Midlands and North West	England	not available	Unknown
Clark	male	Midlands and North West	England	not available	“Used to”
Catherine	female	Midlands and North West	England	1	Yes
Dean	male	Midlands and North West	England	7	Yes
Graham	male	Midlands and North West	England	7	No

^a^SIMD, Scottish Index of Multiple Deprivation; IMD, Index of Multiple Deprivation. SIMD (http://www.gov.scot/Topics/Statistics/SIMD/SIMDPostcodeLookup/ScotlandPostcodeLookup) and IMD (http://imd-by-postcode.opendatacommunities.org/ found using postcode search. SIMD refers to the year 2012, while the IMD refers to 2015. Recent or very old postcodes did not retrieve SIMD details in three cases (hence the missing data). Deciles were used instead of quintiles as the latter were not available for England

^b^1 = most deprived; 10-least deprived

Interviews lasted approximately 30 min and participants have been given pseudonyms to protect their identity. Core themes were common to almost all of the interviews.

Themes identified from non-responder interviews are: s*creening and the wider health care environment, expected role of the PCP* and *responses to the proposed intervention.*

### Screening and the Wider Health Care Environment

Participants demonstrated some knowledge of bowel cancer symptoms and screening, although they reported the need for further awareness-raising and, for the most part, more open discussion of bowel screening to remove stigma. At odds with their actual screening behaviour, interviewees were largely supportive of screening (as well as engaging in discussion on the topic during their consultation), believing it to be important and effective in diagnosing bowel cancer at an earlier stage,

*“Well I think it’s positive, I’m pretty much in favour of health screening although I am aware of some of the arguments against it but no I think that sort of early identification of problems has got to be a good thing.”*

*(Andrew, Lothian)*

Interviewees also viewed bowel screening as part of a holistic approach to their care and demonstrated appreciation of their wider health needs being taken into consideration,

*“I went in about something and he raised a couple of other things, […] right how much alcohol do you drink, things like that. And that felt good. It felt like, you know, you’re there about your health but somebody is thinking about it in a slightly wider, more holistic sense.”*

*(Angela, Lothian)*

Participants reported variable contact with primary care, with a mix of those consulting regularly for long term conditions to those who visited their GP ‘once in a blue moon’. However, participants suggested that the endorsement of their trusted GP would add credibility to the information they were being communicated,

*“It would be a good idea because that then strikes home on the medical side, it reinforces that it’s a health issue. I go to the doctors pretty regularly for blood tests and cholesterol tests and if he was saying to me have I done it, well maybe I should be doing it or even offered me the kit to do it then it would maybe reinforce it.”*

*(Gregor, Lanarkshire)*

### Expected Role of the PCP

With the exception of two participants who reported damaged relationships, interviewees valued their PCP being involved and there was an expectation that they would consider their overall health and wellbeing,

*“I think the more joined up, coordination in the NHS the better. So I don’t see that as an issue, I see that as almost part of wellbeing.”*

*(Graham, Midlands and North West)*

Many participants reported that they would welcome a proactive and fairly hard line approach from their PCP in promoting bowel screening,

*“It should be encouraged a lot more by the GPs encouraging them.”*

*(Bill, Lothian)*

*“You know, more in your face kind of thing.”*

*(Joyce, Lanarkshire)*

Participants also reported that making a personal commitment to their PCP meant they were more likely to perform a particular behaviour,

*“If I made that promise to the GP, yeah, I’ll do it, you’re more likely to send it back to whoever it is.”*

*(Fred, Lothian)*

### Responses to the Proposed Intervention

When asked to consider how they would hypothetically respond to the proposed intervention, participants were broadly supportive of it and felt that it would fit well with their expected care.

Participants noted the primary care consultation as a good opportunity to engage with people about bowel screening, suggesting it was a good chance to ‘seize the moment’ despite being there for another reason,

*“Actually I’m type two diabetic so I’m there seeing the practice nurse as well so questions like that wouldn’t bother me.”*

*(Gavin, Greater Glasgow & Clyde)*

Participants were in favour of the proposed approach and language used,

*“It never is intrusive when it comes to your health, but most GPs know…they deal with the public all the time. […] I think it’s non-threatening what you’ve said.”*

*(Catherine, Midlands and North West)*

They emphasised the need for sensitivity and an avoidance of attributing blame,

*“I think you’ve got to be aware of how you approach a person over anything as delicate as that, that’s down to a doctor’s demeanour.”*

*(Clark, Midlands and North West)*

Participants reported that receiving a leaflet would be good to reinforce the message but a few cautioned against the use of unnecessary words or paperwork, and were unsure whether they would use the implementation intentions.

Overall, interviewees conveyed that the intervention was a good opportunistic approach and went further to suggest handing out kits directly through general practice or using other technology such as text messaging to engage with non-responders.

## Discussion

### Summary

Theoretical concepts of behaviour change, together with the evidence base on bowel screening and complex interventions, were used to develop a brief intervention for opportunistic use in engaging with bowel screening non-responders in primary care. This approach was validated through consultation with stakeholders and bowel screening non-responders.

The professional stakeholders were supportive of the approach and considered it an effective tool to contribute to an increased role for primary care to support participation in bowel screening. They predicted that the intervention acceptance would be variable and adaptations would be made to suit each practice. The planned intervention, including discussions with practices on how to implement it, were therefore modified slightly to allow for greater flexibility when delivering it in practice.

**Fig. 3:**
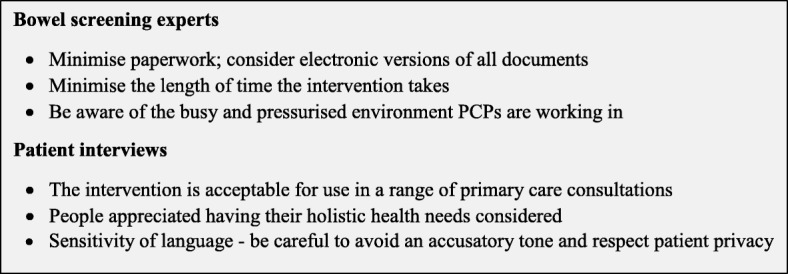
Consultation interviews – main recommendations

Bowel screening non-responders were similarly supportive of the proposed intervention and indicated that they expected and appreciated a broader view of their health. Interviewees were happy to have the topic of screening raised while visiting their GP for another reason, suggesting the intervention is acceptable for use in a number of different consultation scenarios. Interviewees favoured a proactive approach to promoting their participation in bowel screening. Figure 3 summarises the main recommendations for developing the intervention that were subsequently incorporated into the finished product.

### Strengths and limitations

This was a small scale study focusing on providing a theoretical and evidence-based platform for developing an intervention and obtaining feedback on its acceptability.

Non-responder interviewees were a self-selecting group; they indicated support for the screening programme and it is possible those strongly opposed to screening may have not responded to the invitation to participate.

Screening expert interviewees were chosen to access the views of those working at the interface between public health, screening and primary care. One GP was included at this stage. However, further PCP views were obtained when testing the implementation of the intervention in general practice; these are reported separately [23].

There was a low response rate for non-responder interviews so that some perspectives were likely not included. However, key common issues arose in this hard to reach group. This is a sensitive topic area and these recruitment difficulties are encountered in similar studies [24].

### Advantages of our development process

Using a theory and evidence-based approach informed the development of a brief intervention to influence bowel screening uptake. Adopting a multi-stage development method allowed the identification of potential bottlenecks when implementing the intervention and provided scope to make changes, such as adding an email address as a way to request a new test kit. Furthermore, discussion of the proposed intervention during interviews provided a forum for consideration of the real world applicability of the intervention.

### Comparing patient and provider views

Both stakeholders and non-responders reported similar views that informed the ethos of the intervention: their belief in bowel screening, the need for further promotion to raise awareness of screening, and the role of PCPs in adopting a holistic view of patient care; within a model of primary care reflecting a health promoting approach.

Although non-responders’ positive view of bowel screening was seemingly at odds with their behaviour, reported barriers to bowel screening are rarely related to a negative view of screening per se; more so to practical barriers or relative priority [19, 25]. This finding also reflects the evidence for the gap between intention and behaviour [26], with scope for these barriers to be explored between the PCP and the patient during the intervention. Participant reported barriers to bowel screening participation reflect those found elsewhere in the literature, including barriers such as practical issues with completing the test; embarrassment, privacy and competency concerns; fear, and busy lives mediating the intention to complete the test. These barriers are reported in more detail separately [23].

Non-responders in this study referred to PCPs adopting a proactive or even hard line approach to encouraging bowel screening participation. This raises the question of how people use information to make informed decisions and the extent to which it actually mediates their behaviours, an issue addressed elsewhere in the literature [27]. The finding also reinforces the evidence for the weight placed on an NHS recommendation to participate in screening in addition to full disclosure of information [28, 29].

## Conclusion

Developing and refining a complex intervention based on relevant evidence and theory provides a robust product and increases confidence in its implementation and suitability for evaluation in feasibility study. Feedback from potential users of the intervention provides further insights to its real world applicability and inform feasibility testing that predicates its effective delivery in practice [23].

## Additional files

Additional file 1: Intervention bottlenecks – Identified barriers to implementing the brief intervention. (PDF 117 kb)

Additional file 2: Theoretical underpinnings – Details on the theories used to inform the development of the brief intervention. (DOCX 27 kb)

Additional file 3: Screening co-ordinators interview schedule – Interview topic guide for bowel screening stakeholders. (DOCX 16 kb)

Additional file 4: Non-responder interview schedule – Interview topic guide for patient non-responders eligible for the brief intervention. (DOC 29 kb)

## Abbreviations

FOBt: Faecal occult blood test
GP: General Practitioner
NHS: National Health Service
PCP: Primary Care Provider
SBSC: Scottish Bowel Screening Centre
UK: United Kingdom
