# Supplementary figures and images for: Development of an evidence-based brief ‘talking’ intervention for non-responders to bowel screening for use in primary care: stakeholder interviews
Source: BMC Fam Pract. 2018 Jun 30;19:105. doi: 10.1186/s12875-018-0794-6 (PMC6026505; doi:10.1186/s12875-018-0794-6)

## Intervention

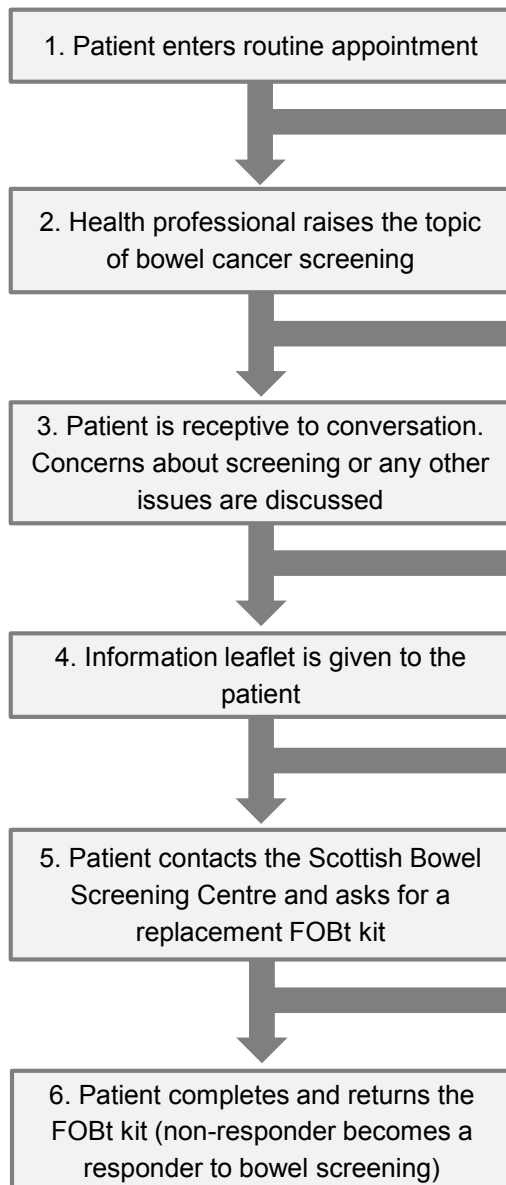

## Potential bottlenecks

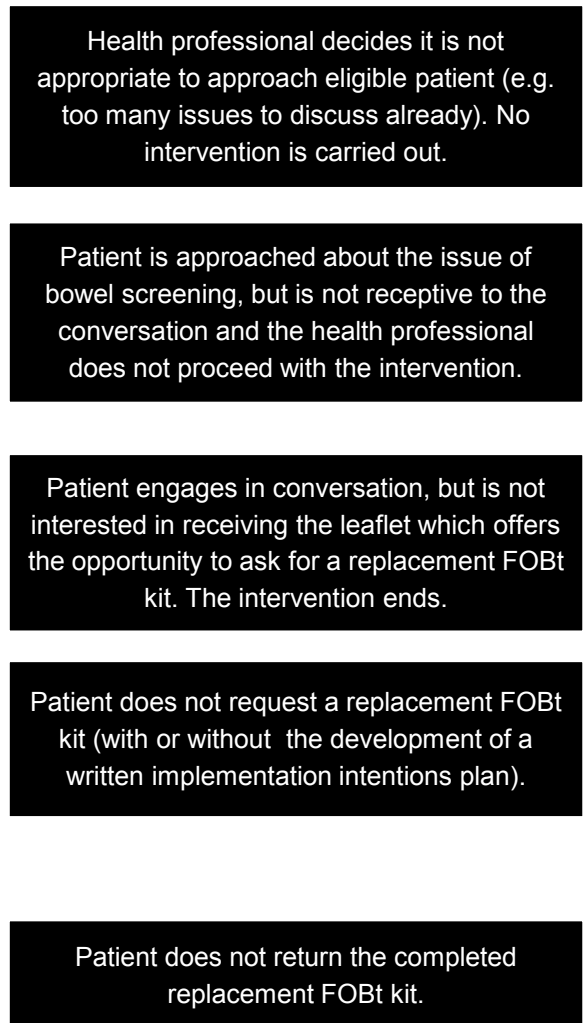

Supplement: Supplementary file 1 — Intervention bottlenecks – Identified barriers to implementing the brief intervention. (PDF 117 kb) [file 12875_2018_794_MOESM1_ESM.pdf]
