# Supplementary material for: Development of an evidence-based brief ‘talking’ intervention for non-responders to bowel screening for use in primary care: stakeholder interviews
Source: BMC Fam Pract. 2018 Jun 30;19:105. doi: 10.1186/s12875-018-0794-6 (PMC6026505; doi:10.1186/s12875-018-0794-6)
Supplement: Supplementary file 2 — Theoretical underpinnings – Details on the theories used to inform the development of the brief intervention. (DOCX 27 kb) [file 12875_2018_794_MOESM2_ESM.docx]

### The implementation literature

Implementation research concerns methods to improve the practical application of research findings [1]. The ‘Behaviour Change Wheel’ [2] provides a comprehensive implementation framework. Three pre-requisites of change interact with and mutually influence the desired health behaviour: motivation (internal and external sources directing behaviour), capability (an individual’s psychological and physical capacity to engage in the intervention) and opportunity (external factors facilitating the behaviour). The intervention sought to address each of these three variables: patient motivation for screening, patient capability to participate in screening and patient opportunity to undertake screening.

### Motivational Interviewing

Motivational interviewing features a patient-centred counselling style to help patients explore and deal with their ambivalence regarding behaviour change [3]. In a process of negotiation, the patient reflects upon the benefits and costs involved. Motivational interviewing has been shown to increase bowel screening participation [4, 5]. Drawing on this approach, our intervention adopted open, non-directive statements to introduce the topic of bowel screening such that the patient chooses whether or not to engage in conversation, for example, “Am I right in thinking that you have not been screened for bowel cancer in the last two years?”

### The Health Behaviour Framework

The Health Behaviour Framework [6] is a synthesis of several health behaviour models, providing a multi-dimensional approach to understanding motivation for behaviour change. The framework has been successfully applied to cancer screening behaviours [7-9]. It considers the context in which interventions are introduced, including service provider characteristics, the health care setting, and larger community and societal influences. Adopting this framework shaped one core aim of the intervention: targeting barriers to screening including lack of knowledge (“Never heard of it”), behavioural response costs (“I felt disgusted”), and self-efficacy (“I tried but I couldn’t do it”).

### Implementation Intentions

Gollwitzer proposes that progress towards a particular goal such as participation in screening requires a deliberative or motivational phase; an evaluation of the costs and benefits of pursuing a goal or behaviour to establish a ‘goal intention’. Following this, a specific plan for implementing the goal intention is made [10]. These plans specifying when, where and how the desired behaviour will be carried out are called implementation intentions. Interventions based on implementation intentions theory have been associated with higher participation in studies of cervical [11], breast [12] and bowel [13] screening. Implementation intentions theory can underpin interventions targeting completion and return of screening tests, offering solutions to emotional and behavioural barriers. Accordingly, our intervention incorporates an implementation intention plan (e.g. “If I get home from work before 5pm then I will call the bowel screening centre to request a kit.”) to be introduced by the health professional (“We find that when we make a plan, it is easier to get it done.”)

### Guidance on complex interventions, behaviour change and informed choice

We followed MRC guidance to inform the development of a multi-factorial, patient-centred complex intervention, considering theoretical underpinnings and using these to shape the design and meet planned outcomes [14]. Similarly, we consulted NICE guidance on behaviour change that highlights social, psychological and cultural determinants of the target behaviour: in particular, we drew on evidence for the acceptability of a planned intervention, and promoting setting and recording behaviour change goals using implementation intentions [15].

Guidance developed as part of the former NHS Cancer Screening Programme was central to the development of the intervention and was reflected in the inclusion of open and non-coercive statements or questions [16]. Achievement of adequate uptake rates should involve informed choice and this should be inherent to decision-making about cancer screening [17].

References

1. Eccles M, The Improved Clinical Effectiveness through Behavioural Research Group (ICEBeRG): Designing theoretically-informed implementation interventions. *Implementation Science* 2006, 1(1):1-8.

2. Michie S, van Stralen MM, West R: The behaviour change wheel: A new method for characterising and designing behaviour change interventions. *Implementation Science* 2011, 6(1):1-12.

3. Treasure J: Motivational interviewing. *Advances in Psychiatric Treatment* 2004, 10(5):331-337.

4. Ling BS, Schoen RE, Trauth JM, Wahed AS, Eury T, Simak DM, Solano FX, Weissfeld JL: Physicians encouraging colorectal screening: a randomized controlled trial of enhanced office and patient management on compliance with colorectal cancer screening. *Archives of internal medicine* 2009, 169(1):47-55.

5. Lasser KE, Murillo J, Medlin E, Lisboa S, Valley-Shah L, Fletcher RH, Emmons KM, Ayanian JZ: A multilevel intervention to promote colorectal cancer screening among community health center patients: results of a pilot study. *BMC Family Practice* 2009, 10(1):1-7.

6. Bastani R, Glenn BA, Taylor VM, Chen MS, Jr., Nguyen TT, Stewart SL, Maxwell AE: Integrating theory into community interventions to reduce liver cancer disparities: The Health Behavior Framework. *Preventive medicine* 2010, 50(1-2):63-67.

7. Tu SP, Yip MP, Chun A, Choe J, Bastani R, Taylor V: Development of intervention materials for individuals with limited English proficiency: lessons learned from "Colorectal Cancer Screening in Chinese Americans". *Medical care* 2008, 46(9 Suppl 1):S51-61.

8. Jo AM, Maxwell AE, Wong WK, Bastani R: Colorectal cancer screening among underserved Korean Americans in Los Angeles County. *Journal Of Immigrant And Minority Health / Center For Minority Public Health* 2008, 10(2):119-126.

9. Maxwell AE, Bastani R, Crespi CM, Danao LL, Cayetano RT: Behavioral mediators of colorectal cancer screening in a randomized controlled intervention trial. *Preventive medicine* 2011, 52(2):167-173.

10. Gollwitzer PM: Goal Achievement: The Role of Intentions. *European Review of Social Psychology* 1993, 4(1):141-185.

11. Sheeran P, Orbell S: Using implementation intentions to increase attendance for cervical cancer screening. *Health Psychol* 2000, 19(3):283-289.

12. Increasing Attendance at Breast Cancer Screening: Field Trial. Final Report to NHSBSP

13. Greiner KA, Daley CM, Epp A, James A, Yeh H-W, Geana M, Born W, Engelman KK, Shellhorn J, Hester CM *et al*: Implementation Intentions and Colorectal Screening: A Randomized Trial in Safety-Net Clinics. *American Journal of Preventive Medicine* 2014, 47(6):703-714.

14. Developing and evaluating complex interventions: new guidance [<http://www.mrc.ac.uk/documents/pdf/complex-interventions-guidance/>]

15. NICE: Behaviour change: general approaches. In*.*: National Institute for Health and Care Excellence; 2007.

16. Consent to Cancer Screening. Cancer Screening Series No 4. [<http://www.cancerscreening.nhs.uk/publications/cs4.pdf>]

17. Jepson RG, Hewison J, Thompson A, Weller D: Patient perspectives on information and choice in cancer screening: a qualitative study in the UK. *Social science & medicine (1982)* 2007, 65(5):890-899.
