# Supplementary material for: Development of an evidence-based brief ‘talking’ intervention for non-responders to bowel screening for use in primary care: stakeholder interviews
Source: BMC Fam Pract. 2018 Jun 30;19:105. doi: 10.1186/s12875-018-0794-6 (PMC6026505; doi:10.1186/s12875-018-0794-6)
Supplement: Supplementary file 3 — Screening co-ordinators interview schedule – Interview topic guide for bowel screening stakeholders. (DOCX 16 kb) [file 12875_2018_794_MOESM3_ESM.docx]

**A primary care brief intervention for bowel screening**

**Interview topic guide: Screening coordinators**

The guide will be used in a flexible and responsive manner, allowing participants to introduce new areas for discussion.

Thanks for participation, brief reminder of the purpose of the study, ensure signed consent form has been received and participant has no remaining questions.

General

What is your role in the delivery of screening in your area?

What impact do you feel, if any, that the Detect Cancer Early (DCE) campaign has had?

Have you been involved in increasing uptake of bowel screening in your area in relation to the DCE campaign?

Are you aware of or involved in any other local initiatives to increase screening?

Do you have any views about what else could be done to increase uptake of screening?

Do you think primary care has a role to play in bowel screening?

If so, what? What do you think the challenges of this are?

Have you been involved with interventions to increase uptake of bowel (or other) screening in primary care?

Can you give details on how these worked or what aspects didn’t work and why?

Do you have any other views on what could be done to increase uptake of screening in the UK or more generally?

Brief Intervention

Talk through the questions one by one

- is wording acceptable? Intrusive?

- other questions that should be asked?

Based on your experience do you think this type of discussion/ intervention is one that GPs/practice nurses would be comfortable having with patients when they were consulting / at the surgery for a different reason?

(Probe whether professionals view this as intrusive, see it as just part of their job, etc)

Would this type of intervention work in a primary care setting?

In what types of routine consultations would this type of conversation be acceptable?

In what type of routine consultations would this type of conversation NOT be acceptable?

Explore any other concerns about the intervention (more pressing issues in patients’ lives, the amount of time that might be required, raising false alarm, worried well, etc.)

Thank participant(s) again, ask if any questions; ask if would like to receive summary of project results.
