# Supplementary material for: Development of an evidence-based brief ‘talking’ intervention for non-responders to bowel screening for use in primary care: stakeholder interviews
Source: BMC Fam Pract. 2018 Jun 30;19:105. doi: 10.1186/s12875-018-0794-6 (PMC6026505; doi:10.1186/s12875-018-0794-6)
Supplement: Supplementary file 4 — Non-responder interview schedule – Interview topic guide for patient non-responders eligible for the brief intervention. (DOC 29 kb) [file 12875_2018_794_MOESM4_ESM.doc]

**A primary care brief intervention for bowel screening**

**Interview topic guide (telephone interviews)**

The guide will be used in a flexible and responsive manner, allowing participants to introduce new areas for discussion.

Thanks for participation, brief reminder of the purpose of the study, ensure signed consent form has been received and participant has no remaining questions

Can you tell me how many invitations you had to take part in bowel cancer screening? Have you ever returned the test kit? If yes, how many times?

(For women) Have you attended breast or cervical screening?

(Probe about attitudes to screening in general)

On the occasions when you decided not to attend bowel screening, what were the reasons?

(Probe for intended and actual response, worries/fears, understanding, views of importance, emotional responses such as embarrassment, time, motivation, other reasons)

Do you think more people should be encouraged to come for bowel screening?

(Probe about perceptions of potential benefits/disadvantages of screening)

Do you have any suggestions for encouraging people to attend screening?

(Probe about type of information, advertising, health professional roles)

As you know, we are trying to develop a short discussion format for GPs and practice nurses to talk to people about taking part in bowel screening

Talk through the questions one by one (no more than 5)

- is wording acceptable? Intrusive?

- other questions that should be asked?

What would be your response if a GP or practice nurse asked you about taking part in bowel screening when you were there for a different issue?

(Probe whether annoyed at the issue being raised, grateful for the chance to ask questions, see it as just part of their job, etc)

We are planning that the GP/practice nurse would give a half-page leaflet to people about how to get another kit if they want one

- what would you think if you were offered this?

Thank participant again, ask if any questions; ask if would like to receive summary of project results; remind participant if s/he has any health concerns to contact her/his GP
